# The Transactions of the American Medical Association

**Published:** 1858-01

**Authors:** 


ART. VI.— *The Transactions of the American Medical Association. Instituted* 1857. *Vol. X.*

Our national medical association has now completed its tenth year; that it has not accomplished all the good which was desired and expected by those who were so zealously interested in its organization, is sufficiently well known; but that it has been nevertheless of great value to the profession, seems to us to be equally true. We conceive that the delegates would have been more numerous, and that the profession would have been more equally represented from all parts of the United States, if the place of meeting had been more accessible, and more interesting to strangers from attractions not connected immediately with the meeting of the association. Of all places in which it would be likely to meet, Nashville is the most inaccessible; but still after once getting there, the characteristic open hand, open heart, and open house, of our friends who inhabit that section of the country, which we ourselves experienced for some time, could not fail to make the few days which were passed by the members in Nashville, as agreeable, if not more so, than they would have been in a large city. All of our friends who attended the last meeting, have been loud in their praises of the good things and good feeling which greeted them on their arrival at Nashville.

The association met at 11 o'clock, May 5th, Dr. Zina Pitcher, of Detroit, in the chair, who was warmly welcomed by Dr. C. K. Winston, in a short but pertinent and beautiful address. The roll of delegates was then called, and a recess taken for the organization of a committee on nominations, who nominated: for president, Paul F. Eve; vice-presidents, R. I. Breckenridge, of Ky., D. M. Reese, of N. Y., W. H. Byford, of Indiana, and H. F. Campbell, of Georgia.

The officers were then conducted to their places by Drs. Wiston, Arnold, and McGugin.

Nothing new or interesting appears in the minutes of the meeting, with the exception of a protest entered by four members of the association, against the admission of delegates from the Oglethorpe Medical College, of Savannan, on the ground that since the existence of that institution, the several chairs of the college have not been regularly filled by the gentlemen appointed to them; this was referred to a committee appointed by the chair, who reported that the college had not in any way violated the letter of the law of the association. Resolutions embodying a plan for the charters of State and County Medical Societies, were offered and adopted; and the association recognized the presentation of a pamphlet by Dr. Campbell, claiming "Priority in the Discovery and Naming of the Excito-Secretory System of Nerves," a matter which has lately been before the profession and an acknowledgment of which was publicly made by Dr. Hall before his death.

The great bulk of the transactions is occupied by the papers referred to the committee on publications. Many of these are articles of great interest, forming by no means a less interesting collection than we have had in the preceding volumes of the transactions.

First, we have the address of Dr. Pitcher, the president. Every medical society is opened by an address on some subject connected with medicine. The opening and closing of all our medical schools is signalized by a similar event, and some of our colleges even go so far as to have a distinct introductory delivered by each professor. Dr. Pitcher's address was short, but in other particulars resembled closely the numerous discourses which are received by us every day, delivered by some professor before some of our colleges or at the meeting of some of our societies: having no particular subject and incidentally alluding to some of the benefits arising from the organization of the association.

We next have a report on the Topography and Epidemics of Maryland. Epidemics of dysentery have appeared during the summer months in various parts of the state, but they have been exceeding mild and managable; the author having had a hundred cases under his own care, two of which only, proved fatal. Malarial fevers, the author finds to be universally under the control of quinine, which is almost the only remedy used in its treatment. In looking through the volume we notice reports on the topography and epidemics of Georgia and Washington territory, which possess more local than general interest, and which we must therefore dismiss with a mere mention.

*Report on Infant Mortality in large cities, the Sources of its Increase and Means for its Diminution.* By D. MEREDITH REESE, M. D., LL. D., etc.

In as much, as at least two copies of this report, extracted from the transactions, have been sent to the Journal, we conclude that the writer desires his scheme for the reduction in the rate of infant mortality to be extensively known.

Dr. Reese in the first place, shows by mortality statistics of the city of New York, that for the last fifty years, forty-nine per cent. of the entire mortality, has been of children under five years of age, and that "still-born and premature birth" interments equal *one-fifth* of the entire infant mortality. The Dr. then assumes that the Creator did not intend that so many individuals should be ushered into the world merely to perish before they arrived at the age of five years. Of course as humane men and physicians, it is our duty to save life whenever we are able, but we should never question the ways of the Almighty; did not our Saviour say, "suffer little children to come unto me, and forbid them not, for of such is the kingdom of Heaven," and it is certainly a question which arises to us, when we contemplate some of our fellow-men; would it not have been better not for themselves and especially for the world, if Providence had pleased that they should have been among those who died young? The measures which the Dr. recommends for the prevention of this horrible mortality, are:

"1. No marriage should be permitted between parties, until the physical health of both has been subjected to professional scrutiny. And such alliances should be prohibited by law, to those of either sex, who are the subjects of those diseases which are known to be hereditary or transmissible to offspring, or such as are fatal to infantile existence. Celibacy should be required by statute of all consumptive, scrofulous, scorbutic, gouty, insane, intemperate, and especially syphilitic individuals of either sex, and this, for grave reasons of state, which concern the public weal. Nor will any course short of such legal prohibition of marriage, adequately correct the evil of that large proportion of infant mortality thus engendered."

The first part of this first proposition would be quite remunerative to the physician, but we fear that it would occupy so much time to examine the condition of marriageable persons, that we would be entirely unable to attend to the sick children; so that this would balance the good which we might do by prevention. The passage of a law by which celibacy should be required of all individuals laboring under the above mentioned peculiarities, would, we conceive, be hardly practicable; in the first place we apprehend that such persons would constitute a majority in most of our legislative bodies; and if, for sake of argument, such a law should be passed, the result would be a great want of increase on the part of our population, or rather, what is more probable, a community composed mostly of illegitimate children; especially as in the second proposition he says, that

"2. To remove the temptations to the unnatural crime of abortionism, and prevent the abandonment and cruel murder of unborn and newly-born infants, among the vicious and depraved portion of our population, for purposes of concealment, as in the case of the illegitimate offspring of shame, foundling hospitals should be provided by the State, in all our large cities, for the reception of infants, and the concealment of the shame of unhallowed mothers, and the protection and preservation of the infant innocents, who are doomed to abandonment by the guilty authors of their being. This, with a proposal for a lying-in asylum for women generally, married or unmarried, 'irrespective of character,' with ordinary sanitary regulations, constitute the bulk of Dr. Reese's suggestion towards the "means of diminishing infant martality." We have extracted enough to give our readers an idea how feasable and intelligent his views are.

*Report on the Medico-Legal Duties of Coroners.* By ALEXANDER J. SEMMES, M. D.

In many cities the office of coroner by no means holds the rank to which it is entitled; any one is thought fit for a coroner, and the office often devolves on men who are entirely incompetent. *The coroner should always be an intelligent physician.* We are fortunate in this city to have this office filled from our number, and hope that this custom will prevail here hereafter. We see by the paper of Dr. Simmes, that in ancient times this office was held in high estimation; and when we reflect upon the importance and responsibility of his duties, we wonder that it has not as much consideration at the present day. The committee on this subject do not report a very flattering account of the usual mode of conducting coroner's inquests in this country; and they we have no doubt are generally correct. A point strongly recommended in the report, is privacy in conducting inquests, at the discretion of the coroner; this we conceive should always be attended to, if for no other reason than to prevent misrepresentations in regard to evidence, and to abate public excitement. The report also recommends a provision for the reasonable remuneration of medical expenses for examination of the body and testimony.

We pass over several interesting reports, to the Report on Deformities after Fractures, by Prof. HAMILTON.

This is the third part, and the conclusion of the report. Dr. Hamilton has of late years associated his name forever with the great subject of fractures and dislocations, and we are very much mistaken if in years to come, there will not be many a young surgeon who will bless the memory of Dr. Hamilton when he is sued for a short leg or a crooked arm, and can turn to the cases collected by the author of this report, and show the *results* of such injuries. If this be not the case, it will be because these reports will have abolished all unjust suits for malpractice. In the first part of this report, which appeared in 1855, fractures of the bones of the head, face, and fractures of the clavicle are considered. The second part, 1856, treats of fractures of the scapula and superior extremity; and the third and last part, of fractures of the remaining bones of the skeleton, with the exception of the bones of the trunk and the bones of the foot, the chapters relating to which were withdrawn by the author with a view to the diminution of the size of the report.

The subject of fractures is by far the most important in the whole range of surgery; first on account of the occasional difficulty in their diagnosis and treatment, and second, because they are injuries which come under the care of every practitioner, and in the county especially, it is not possible to avoid taking charge of the case, no matter how obscure and difficult. Then again, there are certain fractures which it is impossible to treat with a perfect result, and the almost invariable failure of all our most skillful endeavors to bring about a perfect cure in fractures of the femur especially, makes this the most important surgical bone in the skeleton.

Dr. Hamilton has collected, and analyzed with reference to the relative frequency of fracture in different parts of this bone, the age of the patient, the causes, the must successful mode of management, and especially the resulting deformity, one hundred and five cases of fracture of the femur; and for all practical purposes this number is as useful as one hundred thousand. The result of Dr. Hamilton's observations, is that the femur constitutes an exception to the other long bones in respect to the most frequent seat of fracture; this bone being most often broken in its middle third, while the others are most frequently fractured in their lower third.

In fractures of the neck, we have an amount of shortening varying from half an inch to two inches, the average being eleven-twelfths of an inch. The average confinement to the bed is four months and two days.

In fifty-six cases of simple fracture of the shaft, the average shortening is half an inch and $\frac{23}{224}$, or a little more than half an inch. "The average shortening in forty-seven simple cases, in which the exact result is recorded, rejecting not only all of those in which any serious form of complication existed, but also all of those which resulted in no shortening, is three-quarters of an inch and 1-47." "In thirty-two simple cases in which the straight splint, with extension, &c., was used, the average shortening was half an inch and 1-33; and in seven simple fractures in which the double inclined plane is known to have been used, the average shortening is three-quarters of an inch and $\frac{1}{8}$, or including in the estimate, all the fractures of the femur, both simple and complicated, known to have been treated by the straight splint mainly, the average shortening is half an inch and $\frac{1}{5}$, or a fraction less than three-quarters of an inch; while in all the fractures known to have been treated mainly by the double inclined plane, the average shortening is one inch and a quarter."

Our readers cannot fail to see immediately the great value of these conclusions; and we must consider that THESE ARE FACTS, not the speculation of an individual, or rough general ideas formed by a surgeon of extensive experience, but an actual analysis of reported cases, which have been treated by various surgeons and the results of which have been personally ascertained by the author in the presence of witnesses. What is the first question which is asked of the surgeon after he has dressed a limb? How long doctor, will it probably be before I can get about? and immediately after this you are asked whether you can make the bone as good as it was before. A young surgeon would be often very much puzzled to answer these questions unless he could refer to the repo t of fractures by Dr. Hamilton, where he could find the *facts* in relation to this matter. In a court of justice, when the result may be ruin, which has been planned by some envious fellow-practitioner, or an ungrateful patient, who is unwilling to pay; where can we find a defence so certain and reliable as this; the results and deformities which usually follow certain fractures in the hands of the best of surgeons?

In concluding our notice of this report, we cannot but express our admiration of the author's intrepidity in thus daring to publish to the world all his cases: those which were not followed by perfect results as well as those which have been; the satisfaction however, of having placed so much useful matter in the hands of the profession is a sufficient reward. We understand that a few persons are under the impression, that the Treatise on Fractures and Dislocations which is in preparation by the author of this report, will embody the *cases* which are reported. The treatise will embody the results to which the author has arrived in his report; but in a volume of that kind it is almost unnecessary to say that the cases will not be published.*

We notice a report by Prof. Dugas, on dislocations of the shoulder, containing a new principle of diagnosis in these accidents. "If the fingers of the injured limb can be placed by the patient or surgeon upon the sound shoulder, while the elbow touches the thorax, *there can be no dislocation;* and if this cannot be done, *there must be a dislocation.*" This report is accompanied by wood-cuts of the skeleton, which establish this point suffici-

* Appended to this report is an account of some of the American inventions and improvements of apparatus.

ently well. We can think of only one thing which might create a difficulty in the universal application of this method. Should the patient's abdomen be excessively protuberent, might not this principle fail; the elbow not going to the lower part of the thorax, but the thorax advancing to the elbow? It strikes us that there might be a possibility of difficulty in this respect.

*Prize Essay. The Excito-Secretory System of Nerves. Its relations to Physiology and Pathology.* By HENRY FRAZER CAMPBELL, M. D., Professor of Special and Comparative Anatomy in the Medical College of Georgia, etc., etc.

The importance of this essay, and the interest which this subject has of late excited, both in this country and Great Britain, would bespeak for it a more extended notice than it is possible to give in this review of the Transactions. Our readers will see that the present number is filled entirely with original material, and several interesting articles have been unavoidably laid over, as well as articles of great interest, which we had intended should appear in our Eclectic Department.

We have carefully perused the essay of Dr. Campbell, as much for our own gratification, as for purposes of review, and we are confident that every one will consider himself sufficiently repaid by as careful a consideration. We are forcibly struck in the first place, by the appropriate motto which heads the essay, "*Observation becomes experiment when used in severe processes of induction.*—VICTOR COUSIN."

The material for this essay, as Dr. Campbell remarks, is sufficiently abundant and has been accumulating in the hands of the experimental physiologist and the thoughtful and accomplished physician for years, and it is due to the author to state, that these materials have been thoughtfully and ably moulded into one of the most interesting and profitable papers of the day. The illustration of this system and the physiological and pathological phenomena on which this theory is based, are, and have been familiar to every one; though the generalization and arrangement of them into a distinct theory, is new, and to Dr. Campbell belongs the exclusive credit which so useful a discovery merits. "Experiment without analysis, (quoting the words of the author) is ever inadequate and unproductive," and Dr. Camppell has proved by the valuable contribution which he has presented to the literature of the nervous system, that careful and judicious induction from the experiments of others is more valuable even than the original observations.

In 1837, Dr. Marshall Hall published his great discovery of the relation which existed between the sensory and the motor portions of the nervous system, called the *excito-motory* functions. Claude Bernard has demonstrated by his experiments in producing artificial diabetes by mechanical irritation of the floor of the fourth ventricle, that the nervous centres may be made to exert an undue influence on secretiòn, by mechanical means. Magendie's experiments on the results of the division of the fifth pair, go to establish the same doctrine; but Dr. Campbell has shown that in addition to the influence which the sensory system has on motion, it has its influence upon secretion; producing normally the effects which are familiar to us as physiologists, and pathologically the effects which were produced by Magendie in his experiments on the fifth pair, and by Bernard in his experiments of irritation, the floor of the fourth ventricle. Illustrations of this fact are familiar to every one; the profuse secretion of tears when the eye is exposed to the cold wind; the modifications in the urinary secretion in diseases of the uterus, of the skin, etc., are occurrences which we meet with every day.

We copy in conclusion, a quotation made by the author, which applies to almost every decided advancement in science. "The ultimate attainment of every truth," write the great Sir William Hamilton, "has ever been by *gradual approach*," and, as it is not unkindred to our theme, we quote his apt illustration: "The history, in regard to the discovery of the distinction between sensitive and motor filaments in the nerves, finally established by Sir C. Bell, was only the last of a long series of *previous observations* tending to the same effect;" or, in the words of Prof. Wherwell, "the facts were known, but they were insulated and unconnected till the discoverer supplied from his own stores, a principle of connection. The pearls are there, but they will not hang together till some one supplies the string."

The concluding article in this volume of the Transactions, is another prize essay by Dr. Wm. A. Hammond, U. S. A., entitled, *Experimental Researches relative to the Nutritive Value and Physiological Effects of Albumen, Starch and Gum, when singly and exclusivelg used as food.*

These investigations were all instituted upon the person of the author, and show an enthusiasm and devotion to science, worthy of the cause. These investigations were commenced by a careful analysis of the quantity and quality of the urine and fæces, the state of the pulse, temperature of the body, etc., during the author's ordinary avocations and while he was taking his usual quantity and quality of food. With this as a point of departure, Dr. Hammond made observations of the effects of an exclusive diet of pure albumen, and water, which he continued for ten days; of an exclusive diet of starch and water, which he continued also for ten days; and of gum and water, which he was compelled to discontinue after the fourth day.

The more important results of the exclusive diet of albumen, were the loss of weight, commencing on the fifth day, and the appearance of albumen in the urine on the sixth day. That life cannot be sustained by a single article of diet, even though it be of so nutritious a character as albumen, has been sufficiently well established even before the experiments of Dr. Hammond; but it has been the general opinion that this diet does not produce albuminuria. The latter fact is established by the author in his own case, where albumen appeared in the urine on the sixth day and increased in quantity to the tenth, when the experiment was concluded. After using an exclusive diet of starch for a few days, sugar appeared in the urine, debility was excessive, with palpitation of the heart and pyrosis. The desire for other food was very great. The author was compelled to discontinue the diet of gum after the fourth day, as it produced great hunger, debility, and febrile movement, together with severe pains in the abdomen, and great loss of weight.

These experiments by Dr. Hammond are extremely useful, demonstrating the inability of a single nutritious element to sustain life, and showing interesting effects of such a course of diet upon the constitution of the urine, fæces and blood. In the case of gum, we may draw an important lesson from these experiments in the administration of this article in disease. It is here shown to be absolutely without nutritious qualities, and moreover to be positively injurious. Under these circumstances we would be disposed to agree with the Dr. in especially condemning it as an article of food for the sick.

We are sorry that our space does not enable us to do better justice to some of the interesting and important papers contained in this volume: especially the papers by Dr. Hamilton, Dr. Campbell and Dr. Hamilton; but we have far exceeded the limits which we had prescribed for ourselves before commencing this review. F.

